# *Nlrc3* Knockout Mice Showed Renal Pathological Changes After HTNV Infection

**DOI:** 10.3389/fimmu.2021.692509

**Published:** 2021-07-16

**Authors:** Ruixue Ma, Xiaoxiao Zhang, Jiayi Shu, Ziyu Liu, Wenjie Sun, Shiyuan Hou, Yunhua Lv, Qikang Ying, Fang Wang, Xia Jin, Rongrong Liu, Xingan Wu

**Affiliations:** ^1^ Department of Microbiology, School of Basic Medicine, Fourth Military Medical University, Xi’an, China; ^2^ Shanghai Public Health Clinical Center, Fudan University, Shanghai, China; ^3^ The College of Life Sciences and Medicine, Northwest University, Xi’an, China

**Keywords:** HFRS, HTNV, NLRC3, renal hemorrhage, renal tubule dilation

## Abstract

Hantaan virus (HTNV) infects humans and causes hemorrhagic fever with renal syndrome (HFRS). The development of well-characterized animal models of HFRS could accelerate the testing of vaccine candidates and therapeutic agents and provide a useful tool for studying the pathogenesis of HFRS. Because NLRC3 has multiple immunoregulatory roles, we investigated the susceptibility of *Nlrc3^−/−^* mice to HTNV infection in order to establish a new model of HFRS. *Nlrc3^−/−^* mice developed weight loss, renal hemorrhage, and tubule dilation after HTNV infection, recapitulating many clinical symptoms of human HFRS. Moreover, infected *Nlrc3^−/−^* mice showed higher viral loads in serum, spleen, and kidney than wild type C57BL/6 (WT) mice, and some of them manifested more hematological disorders and significant pathological changes within multiple organs than WT mice. Our results identify that HTNV infected *Nlrc3^−/−^* mice can develop clinical symptoms and pathological changes resembling patients with HFRS, suggesting a new model for studying the pathogenesis and testing of candidate vaccines and therapeutics.

## Introduction

Hemorrhagic fever with renal syndrome (HFRS) presents acute interstitial nephropathy after zoonotic transmission of hantaviruses from rodents to humans. The disease mainly occurs in Asia and Europe (1, 2). The most prevalent pathogens causing HFRS are Dobrava virus (DOBV), Puumala virus (PUUV), Seoul virus (SEOV), and Hantaan virus (HTNV), each of which causes different severity of HFRS. The disease features are different in North and South America, where Hantavirus infection causes hantavirus pulmonary syndrome (HPS), mainly by Andes virus (ANDV) and Sin Nombre virus (SNV) (3).

HFRS occurs with characteristics of high fever, hypotension, renal failure, and hemorrhage (4). It can be divided into five clinical stages: febrile, hypotensive shock, oliguric, diuretic, and convalescent. An acute episode of HTNV infection leading to HFRS is often accompanied by thrombocytopenia, leukocytosis, anemia, elevated serum creatinine, and liver enzymes (5). These clinical symptoms and signs can partly be explained by the fact that HTNV principally attacks the kidney and induces kidney injury characterized by acute tubulointerstitial nephritis involving infiltration of inflammatory cells (6), consequently afflicting multiple other organs (7).

Rodents are the natural reservoirs for Hantaviruses that persistently infect animals without causing disease (8). Their excreta, such as saliva, urine, and feces, become potential contaminants that can infect humans who inhale small particle aerosols of contaminated excreta. Hantaviruses generally cause asymptomatic infection in adult BALB/c and C57BL/6 mice (9), but lethal neurological disease or persistent infection in suckling and newborn mice (10, 11). Although nephropathic abnormalities were demonstrated in experimentally PUUV infected cynomolgus macaques (Macaca fascicularis), these animals showed only mild proteinuria, immune cell filtration, and tubular damage kidneys, similar to a mild case of HFRS (12). In addition, hamsters infected with HTNV *via* nasal route led to a persistent asymptomatic infection, characterized by sporadic viremia but high levels of viral genome copies in the lungs, brain, and kidneys. Ferrets infected with high dose HTNV *via* muscle injection showed gradual bodyweight decrease (5-12% over time) but intact renal functions, absence of viremia, and lack of virus transmission to organs. In marmosets, intramuscular injection of HTNV led to a high degree of seroconversion and production of neutralizing antibodies; consistently, these animals had no kidney damage, viremia, or virus transmission to organs (13).

Despite their imperfection, animal models using rats and mice were extensively employed to study Hantavirus pathogenesis (14). Yoshimatsu et al. showed that infection of SCID mice with HTNV caused lethal wasting disease with pulmonary edema (15, 16), and the depletion of neutrophils prevented the development of pulmonary edema, but no characteristic hemorrhagic lesions of HFRS were found (15). Shimizu et al. found that HTNV isolated from HFRS patients can infect 6-week-old female BALB/c mice intravenously, causing viremia for 6-9 days, showing signs of disease including temporary weight loss, hair wrinkles, kidney exudation (17). In the same mouse model, there was macroscopic hemorrhage in the border between the renal cortex and medulla, but whether the observed histopathological changes were associated with reduced kidney function had not been examined. In humanized mice, HTNV infection leads to weight loss, decreased activity, ruffled fur, and lung injury, which seem the lung is the main target organs for viral infection (18). Therefore, a suitable animal model of HTNV infection that captures most disease features of HFRS is still needed.

NLRs are the most prominent family of intracellular innate immune receptors and serve divergent functions in regulating innate immunity. Although the best-known NLRs (e.g., NLRP3) exhibits positive regulatory function in inflammatory and immune activation, an unusual sub-group of NLRs, defined as inhibitory NLRs, exhibit inhibitory function in inflammatory responses. To understand the mechanism of NLR-mediated innate immune suppression, ligands for inhibitory NLRs such as NLRC3 and NLRX1 have been examined (19). NLRC3 is a negative regulator that attenuates type I interferon (IFN-I) response by sequestering and attenuating stimulator of interferon genes (STING) activation (19). NLRX1, as a central homeostatic gatekeeper between mitochondrial biology and immunological response, has been implicated in a wide range of diseases, both pathogen-driven and otherwise (20). Because HTNV disease manifests much dysregulation of inflammation and immune activation, we chose *Nlrc3^-/-^* mice to investigate the molecular mechanisms of the disease.

This study demonstrated that *Nlrc3* ^−/−^ mice model resembles systematic injury observed in patients with HFRS, characterized by thrombocytopenia, renal tubule dilation, and hemorrhage. Viral burden analysis revealed that *Nlrc3^−/−^* mice presented higher viral loads in multiple tissues, including serum, spleen, and kidney. Our studies establish a mouse model for HTNV infection useful for evaluating candidate vaccines and therapeutics.

## Methods

### Virus Stocks

HTNV strain 76-118 was donated by Changshou Hang (Chinese Center for Disease Control and Prevention) and then preserved and expanded in our laboratory. Vero E6 cells were used for virus propagation, and infectious titers were quantified using 50% tissue culture infectious dose (TCID_50_) by immunofluorescence assays (IFAs). Conversion between TCID_50_ and Plaque-forming units (PFUs) was based on the following calculation: 1 PFUs ≈0.7×TCID_50_ (21).

### Generation of *Nlrc3* Knockout Mouse Model

Wild type C57BL/6J mice and *Nlrc3* knockout (*Nlrc3^−/−^*) mouse models were designed and developed by Shanghai Model Organisms Center, Inc (Shanghai, China). For obtaining *Nlrc3^−/−^* mouse, Cas9 mRNA and sgRNAs were prepared by *in vitro* transcription. Two sgRNAs targeted to delete exons 2-3 of *Nlrc3* gene: 5’- GTTGGTCTAATAAGCATCCTGGG -3’, 5’- TCCCATGAAACCATGTCAGAAGG -3’. Zygotes of C57BL/6J mice were received and injected with *In vitro*-transcribed Cas9 mRNA and sgRNAs and transferred to pseudopregnant recipients. The genotype of obtained F0 mice was identified and confirmed by PCR and sequencing using primer pairs: F-5’- GTGATGTCTGCTTACC CCGTCTCC -3’; R-5’- GCCTGTGCCGCCTCTCA -3’. By crossing with C57BL/6J mice, positive F0 mice were chosen for obtaining F1 heterozygous *Nlrc3* knockout mice. PCR and sequencing analysis validated the obtained F1 mice. For obtaining the heterozygous *Nlrc3^−/−^* mice, female and male F1 heterozygous mice were intercrossed randomly. Mice were aged 6–8 weeks were used for all studies.

### Animal Infection and Preparation of Samples

A total of 5×10^5^ PFUs of HTNV in 50 ul of media was inoculated through the intraperitoneal route. Mice in control groups received identical amounts of PBS. Twenty to twenty-five *Nlrc3^−/−^* or WT mice were used. Each type of mice was subdivided into five groups for sampling at 0, 3, 6, 9, and 12 days post-infection. Body weights and anal temperatures were recorded daily. At each time point, four to five animals from both groups were euthanized for testing. For virological analysis, fresh tissue samples were collected from the heart, liver, spleen, lung, kidney, and brain and then frozen at -80°C immediately. Mice were perfused with cold PBS, and organs were fixed in 4% paraformaldehyde for 4 hours for histological and immunohistochemical analysis. Blood specimen was collected for viral load testing and blood routine examination, and serum was isolated for biochemistry analysis.

### Cytokine Assay

A total of 25 μL serum from each mouse was assayed for 13 different cytokines with LEGENDplex™ Mouse Anti-Virus Response Panel (13-plex) (BioLegend) according to the manufacturer’s instructions. FACS Calibur cytometer (Becton Dickinson Biosciences, CA) was used for detection. The data were analyzed by LEGENDplex v8.0. Software.

### Real-Time RT-PCR Assay

Tissue samples from each mouse were weighed and immersed in PBS and homogenized by an automated homogenizer-TissueLyser II (QIAGEN, Germany), using Frequency 30/s and Time 5 min. For total RNA extraction, 200 μL tissue homogenates were transferred to 500 μL TRIzol reagent, followed by chloroform extraction and isopropanol precipitation. 140 μL of serum samples were transferred to 560 μL AVL buffer containing carrier RNA (Qiagen, UK) for RNA extraction using the QIAamp Viral RNA Mini kit (Qiagen, UK). Finally, the RNA was redissolved in RNase-free water. An HTNV specific real-time RT-PCR assay was performed for the detection of viral RNA from each sample. The primer sequences specifically targeting for HTNV *s* segment were adopted as follows: F-5’- GATCAGTCACAGTCTAGTCA-3’ and R-5’- TGATTCTTCCACCATTTTGT-3’. One-Step TB Green PrimeScript™ RT-PCR Kit II (Perfect Real Time) (Takara, Japan) was used for the qRT-PCR assay. The final master mix (20μL) comprised 2 μL of RNA, 5.6 μL of RNase Free dH_2_O, 0.8μL of Forward and Reverse primer (10 μM), 0.8 μL of PrimScript 1 Step Enzyme Mix 2, and 10 μL of 2×One Step TB Green RT-PCR Buffer 4. The cycling conditions used were 42°C for 5 minutes, 95°C for 10 seconds, then followed by 40 cycles of 95°C for 0 seconds, 65°C for 15 seconds, and 95°C for 0 seconds. Reactions were run and analyzed on the LightCycler platform (Roche Diagnostics, USA).

### Absolute Quantification of Viral Load

Viral RNA oligonucleotides as RNA standard was obtained by *in-vitro*-transcription from DNA using MAXIscript Kit (Thermo Scientific, UK), which comprised 562 bases of HTNV RNA targeted by the assay (22), and then quantified by an ultramicro microplate spectrophotometer (BioTek Epoch, USA), the concentration of the standard RNA stock was 800 ng/μL. For assessing viral load in each sample, the standard RNA was prepared with 10-fold serial dilution, ranging from 10^10^ copies to 10^3^ copies per reaction. Our standard RNA has a detection limit of 10^10^ copies to 10^1^copies per reaction, and thus we set the threshold of detection for this assay at 100 genome copies per reaction. In-run analysis, standard curves were established with a slope of -3.6624, a Y-intercept of 41.68 cycles, and an R^2^ value of 0.9996.

### Analysis of Protein Levels by Western Blotting

Tissue lysates were prepared from mouse tissue samples. Bicinchoninic acid (BCA) protein assay kit (Thermo Scientific, UK) was used to detect protein concentration. For protein analysis, 30 μg of total protein from each sample was uploaded on SDS-polyacrylamide gel and transferred to NC membranes. Then, 5% skimmed milk solution was used for blocking at room temperature for 2 hr. Next, 1:1000 diluted the 1A8 monoclonal antibody against HTNV-NP (prepared and purified by our laboratory) (23, 24) was added and incubated at 4°C overnight. Finally, membranes were incubated with secondary antibodies, HRP-conjugated Rabbit anti-mouse IgG, or HRP-conjugated Mouse anti-rabbit IgG at room temperature for 2 hr. The chemiluminescent substrate of HRP was then added to detect the proteins. Densitometric analysis of protein band intensity was performing by using Image J software (NIH, USA). β-actin was used as a loading control for normalization.

### Immunohistochemistry Assay

Tissue samples were embedded in paraffin and sliced into 4 mm for immunohistochemistry staining. Sections undergo deparaffinization and rehydration and inactivation of endogenous peroxidases. Sections were subjected to antigen retrieval in citrate buffer (PH=6) at 95°C for 10 min. Then, 5% normal goat serum was used for blocking at room temperature for 40 min. The mAb 1A8 was used as the primary antibody for HTNV NP antigen and incubated with tissue sections at 4°C overnight. Followed by staining with FITC anti-mouse IgG (Invitrogen) at 37°C for 1 hr, and then DAPI for 10 min at room temperature. 3DHISTECH (Hungary) was used for image acquisition and CaseViewer2.4 for data analysis.

### Histology Examination

In order to preserve cell and tissue morphology, 4% neutral buffered formalin was used to fix tissue samples for histopathological analysis. These tissues were processed into 5 mm sections and then stained with hematoxylin and eosin (H&E). Finally, the sections were examined by light microscopy, and the images were captured by 3DHISTECH (Hungary), analyzed by CaseViewer2.4. The degree of lesions was scored visually using a pre-set 4-grades system: within normal limits as 0, minimal as 1, mild as 2, moderate as 3, severe as 4.

### Animal Subject Studies

All animal experiments were approved by the Committee of Laboratory Experimentation of Fourth Military Medical University Animal Center. (No.20210403).

### Statistics

Data were analyzed using GraphPad Prism 9 software. Unless otherwise specified, all data were presented as the mean ± the standard deviation (SD). The *t*-tests or One-way ANOVA was used as specified in the figure legends. A *P*-value of less than 0.05 was considered significant.

## Results

### Detection of Viral Load in Various Organs of *Nlrc3^−/−^* Mice After Intraperitoneal Infection With HTNV

**Figure 1:**
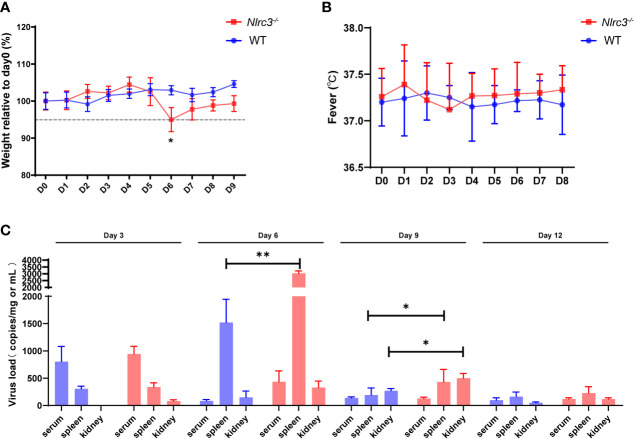
*Nlrc3^−/−^* mice are susceptible to HTNV infection. Mice were intraperitoneally inoculated with 5×10^5^ PFUs of HTNV 76-118. **(A)** Body weights were recorded daily up to 9 dpi for *Nlrc3^−/−^* mice (n=15) and C57BL/6J wild-type (WT) mice (n=10). At the time of inoculation (Day 0), the body weights of mice were set as the baseline, and weights relative to baseline were shown. **(B)** Body temperature was recorded daily up to day 8 post-infection for *Nlrc3^−/−^* mice (n=25) and C57BL/6J wild-type (WT) mice (n=20). **(C)** Viral loads in the serum, spleen, and kidney of HTNV-infected mice were measured on 3, 6, 9, and 12 dpi (*Nlrc3^−/−^* mice, n=25, WT mice, n=20) by real-time PCR. The limit of detection (LOD) was indicated. Bars represented means ± SD. Asterisks indicate a significant difference in the quantity of viral RNA loads (*p* < 0.05).

To characterize the susceptibility of *Nlrc3^−/−^* mice to HTNV infection, a group of *Nlrc3^−/−^* mice were intraperitoneally infected with 5×10^5^ PFUs of HTNV per mouse, using WT mice as controls. All animals were monitored daily for clinical symptoms, temperature, and body weight changes. Mice were sacrificed on 3, 6, 9, and 12 days post-infection (dpi) to analyze various laboratory parameters, viral loads, and pathology. *Nlrc3^−/−^* mice began to lose weight on 4 dpi, and by 6 dpi, Weight loss > 5% of their starting body weight was recorded. In comparison, WT mice exhibited no weight loss (**Figure 1A**). No fever was recorded in both groups (**Figure 1B**).

In order to assess the dissemination and replication of HTNV in viral challenged mice, the serum and major organs (including heart, liver, spleen, lung, kidney, and brain) were collected for absolute quantification of viral RNA copy numbers. Results showed that viremia occurred on 3 dpi in both groups (*Nlrc3^−/−^* mice: 943 ± 140 copies equivalents per mL; WT mice: 803 ± 277 copies per mL). Notably, the serum viral loads of WT mice peaked at 3 dpi and decreased rapidly after that, whereas in *Nlrc3^−/−^* mice, the viral load maintained an intermediate level until 6 dpi and then progressively decreased to the low level on 9 dpi (**Figure 1C**). In addition, we collected the whole blood of HTNV infected mice on days 0, 3, 6, and 9 post-infection to determine the viral replication in blood. The results showed that viral load increased on 3 dpi and then decreased suggesting viral replication in blood cells. As shown in **Supplementary Figure 1**, the viral copies number in *Nlrc3^-/-^* mice was higher than that in WT mice (*Nlrc3^-/-^* mice: 5752 ± 502 copies per mL; WT mice: 3589 ± 869 copies per mL) at 3 dpi. Moreover, 2-fold higher viral loads were detected in the spleens of *Nlrc3^−/−^* mice (3033 ± 175 copies equivalents per mg) than WT mice (1520 ± 425 copies per mg) on 6 dpi; the same pattern was observed in the kidneys on 9 dpi (*Nlrc3^−/−^* mice: 500 ± 87 copies equivalents per mg; WT mice: 270 ± 40 copies per mg) (**Figure 1C**). Overall, modest higher viral loads were detected in *Nlrc3^−/−^* mice than WT mice.

**Figure 2:**
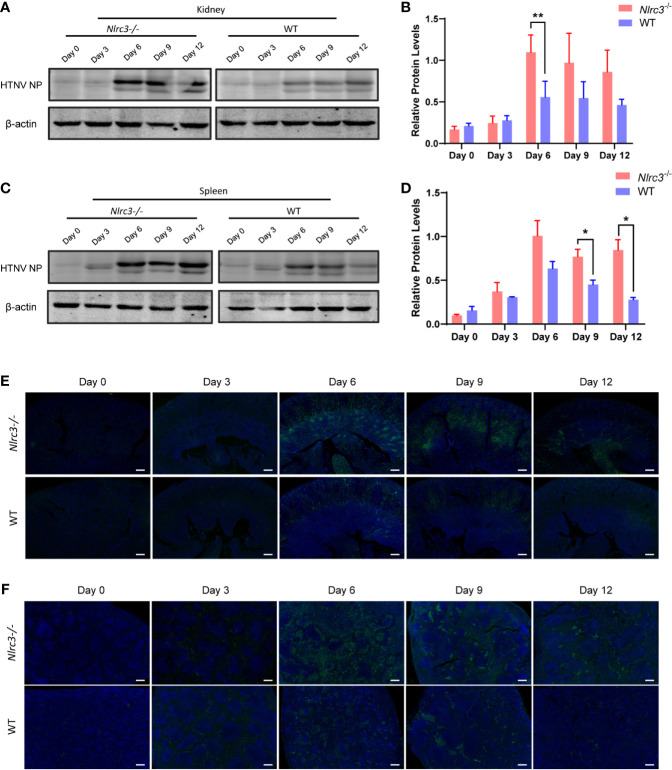
Expression levels of HTNV nucleoprotein (NP) in spleen and kidney samples obtained from *Nlrc3^−/−^* and WT mice after viral challenge. Samples were collected on 3, 6, 9, and 12 dpi for Western blot and immunohistochemistry (IHC) analysis. **(A)** Western blot results of the kidneys of *Nlrc3^−/−^* and WT mice. Lanes represent corresponding time points. **(B)** Densitometric analysis of band intensity for A (*Nlrc3^−/−^* mice, n=15; WT mice, n=10). **(C)** Western blot results of the spleens. **(D)** Densitometric analysis of band intensity for C (*Nlrc3^−/−^* mice, n=25; WT mice, n=20). IHC staining of mouse kidney **(E)** and spleen sections **(F)** to detect HTNV nucleocapsid protein in *Nlrc3^−/−^* (top) and WT mice (bottom) after infection. HTNV NP (green), and DAPI (blue). IHC images are representative of at least three samples. Scale bars, 50 μm. *p < 0.05; **p < 0.01.

Next, we examined the HTNV nucleoprotein (NP) levels. As shown in **Figures 2A, C**, *Nlrc3^−/−^* mice presented higher viral antigen levels than WT mice in the spleen and kidney. Statistical analysis of viral antigen in the kidney and spleen was showed in **Figures 2B, D**, respectively. Low levels of viral antigens were detected in the heart, liver, lung, and brain of *Nlrc3^−/−^* mice (**Supplementary Figure 2**). Viral NP protein levels in the heart, lung and brain of *Nlrc3^−/−^* mice were lower than WT mice on protein level (**Supplementary Figures 2B, F, H**), but the statistical significances were shown between the heart and brain both on mRNA and protein levels (**Supplementary Figures 2A, E, I**). This finding suggested that active viral replication occurred in the blood, spleen, and kidney of *Nlrc3^−/−^* mice after HTNV infection.

Finally, we used immunofluorescence to detect the expression levels of HTNV NP in the spleen and kidney. HTNV NP fluorescence intensity was higher in kidney renal tubules (**Figure 2E**) and spleen red pulp (**Figure 2F**) of *Nlrc3^−/−^* mice than WT mice on 6 dpi and 9 dpi. Together, these results indicate that the *Nlrc3^−/−^* mice are more susceptible to intraperitoneal infection of HTNV.

### The Routine Blood Tests of *Nlrc3^−/−^* Mice After Infection of HTNV

It has been widely accepted that HFRS pathology is largely immune-mediated, including immune complexes, complement activation, T cell response (24–26), and HTNV-induced cytokine production (26, 27), causing multiple organs injury. In addition, thrombocytopenia is a common clinical feature in those infected with viral hemorrhagic fever (VHF) viruses. Thus, we performed a hematology test first using whole blood collected from *Nlrc3^−/−^* and WT mice on 3, 6, 9, and 12 dpi.

**Figure 3:**
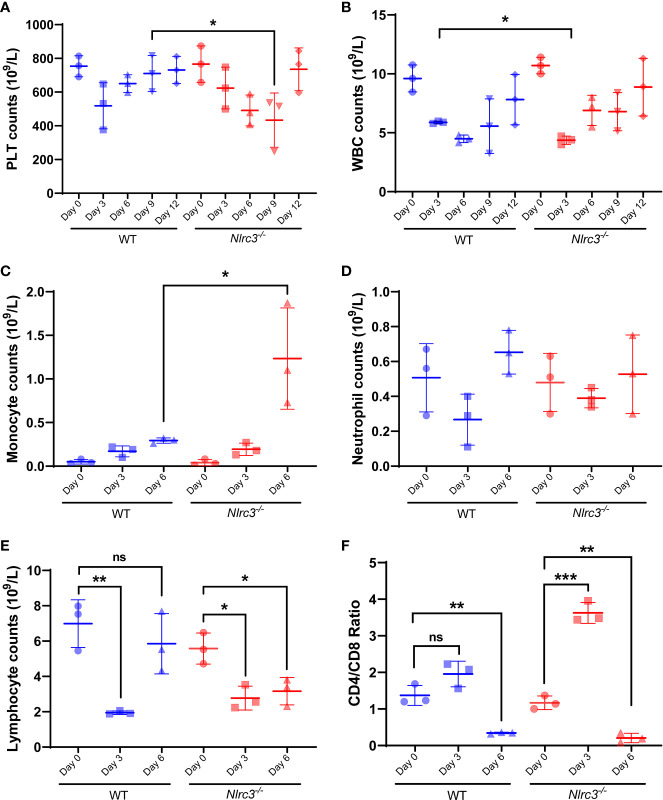
Hematological examination of *Nlrc3^−/−^* and WT mice post-infection. The whole blood was collected on days 3, 6, 9, and 12 post-infection for a routine blood test. The whole blood was centrifuged at 1,500 g for 10 mins at room temperature to obtain serum for biochemical tests. **(A)** Platelet (PLT) counts in the whole blood (10^9^/L); **(B)** White blood cells (WBCs) counts in the whole blood (10^9^/L); **(C)** Monocyte counts in the whole blood (10^9^/L); **(D)** Neutrophil counts in the whole blood (10^9^/L); **(E)** Lymphocyte counts in the whole blood (10^9^/L); **(F)** The ratio of CD4/CD8 in the whole blood; Bars are means ± SD. *Nlrc3^−/−^* mice, n=3; WT mice, n=3. *p < 0.05; **p < 0.01; ***p < 0.001; ns is short for no significance.

Results showed that PLT counts in WT mice decreased on 3dpi and then gradually recovered, but they continued to decrease in *Nlrc3^−/−^* mice until reaching the lowest value on 9 dpi, significantly lower than that in WT on 9 dpi (P<0.05) (**Figure 3A**). The decrease of WBC counts was observed on 3 dpi and then gradually recovered to normal levels in both mouse strains (**Figure 3B**). To further dissect the subset changes in WBCs, we analyzed on 0, 3, 6 dpi the number of monocytes, neutrophils, lymphocytes, and T cells by routine blood tests combined with flow cytometry assay. The number of monocytes increased significantly in *Nlrc3^−/−^* mice on 6 dpi but not in WT mice (**Figure 3C**). The neutrophils in both types of mice decreased slightly but recovered to average level on 6 dpi (**Figure 3D**). The number of lymphocytes decreased significantly on 3 dpi, while they began to rise in WT mice on 6 dpi, the lymphocyte counts remained at a low level on 6 dpi in *Nlrc3^−/−^* mice (**Figure 3E**). The ratio of CD3^+^ CD4^+^ T cells versus CD3^+^ CD8^+^ T cells in peripheral blood was determined by flow cytometry and found to be increased at 3 dpi and then rapidly reversed at 6 dpi, consistent with changes often observed in HFRS patients (**Figure 3F**), in whom CD4^+^ T cells dramatically decreased, and CD8^+^ T cells remain unchanged or compensatory expanded to result in a reversed ratio of CD4/CD8. These findings suggested that intraperitoneal inoculation of HTNV into *Nlrc3^−/−^* mice, to some degree, induced the main clinical features of patients infected with HTNV, such as thrombocytopenia and reversed CD4/CD8 T cell ratio.

### Pathological Changes in the Kidney of Infected *Nlrc3^−/−^* Mice Shows Marked Renal Interstitial Hemorrhage and Renal Tubules Dilatation

**Figure 4:**
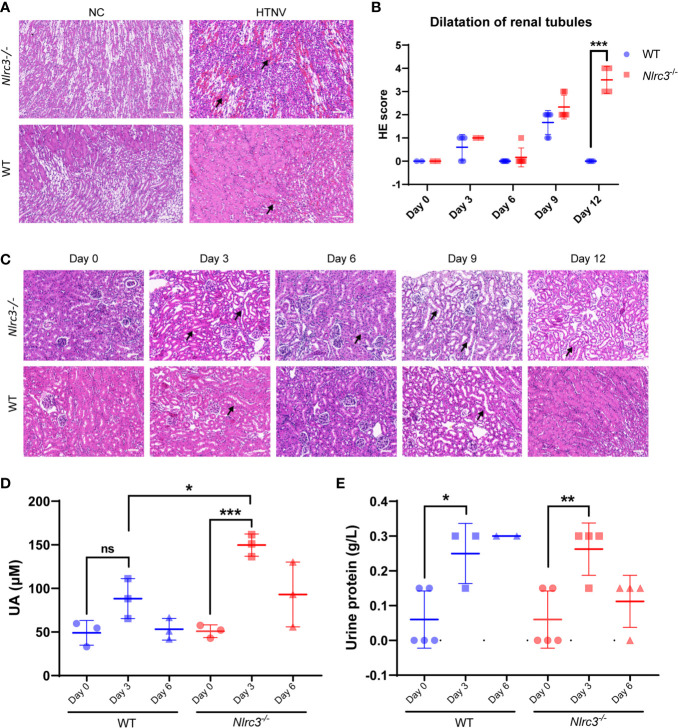
Renal pathological changes in HTNV-infected mice. **(A)** The kidney was collected on 3, 6, 9, and 12 dpi and fixed in 4% paraformaldehyde, succumbed to H&E staining. Pathological analysis showing focal hemorrhage (black arrow) in the renal medulla of HTNV-infected mice on 6 days post-inoculation. **(B)** Statistical figure based on histological scores of dilations of renal tubules for *Nlrc3^−/−^* mice (n=6) and WT mice (n=6). **(C)** Pathological analysis showing extended renal tubules (black arrow) in mice after HTNV infection. H&E images are representative of at least three samples. Scale bars, 50 μm. **(D)** The whole blood was centrifuged at 1,500 g for 10 mins at room temperature to obtain serum for biochemical detection. The levels of uric acid (UA) in the serum (μM) of *Nlrc3^−/−^* mice (n=3) and WT mice (n=3). **(E)** Urine test of *Nlrc3^−/−^* and WT mice post-infection. Urine was collected on days 0 (n=5 in both groups), 3 (*Nlrc3^−/−^* mice: n=4; WT mice: n=3), and 6 post-infection (*Nlrc3^−/−^* mice: n=4; WT mice: n=2) for urine routine examination. The level of urine protein of infected mice was quantified. Bars are means ± SD. *p < 0.05; **p < 0.01; ***p < 0.001; ns is short for no significance.

Next, pathological changes of HTNV-infected mice were evaluated by H&E staining and found to exist in the kidney of all infected *Nlrc3^−/−^* mice manifested as Edema of renal tubular epithelial cells. On 6 dpi, hemorrhagic lesions were observed in the renal medulla (**Figure 4A**). Renal tubule dilatation was observed early as 3 dpi, its severity peaked at 9 dpi and persisted until 12 dpi, and it is more pronounced in *Nlrc3^−/−^* mice than WT mice (**Figures 4B, C**). These findings indicated that acute nephropathy characterized by renal tubules dilatation and renal interstitial hemorrhage was induced in HTNV-infected *Nlrc3^−/−^* mice. As to other pathological changes in the liver, spleen, lung, these two groups of mice showed similar results. These mainly included extra-medullary hematopoiesis in the spleen, alveolar wall thickening in the lung, and liver degeneration (**Supplementary Figures 3A–F**). Based on the above, the pathological features observed in the kidney of *Nlrc3^−/−^* mice during the acute phase of HTNV infection closely resembled the clinical-pathological changes in human HFRS cases.

Based on this, we further examined the related indexes of blood and urine in mice for functional disorders of the kidney. Mouse sera were collected for assessment of alanine aminotransferase (ALT), creatine kinase MB fraction (CK-MB), urea, as well as uric acid (UA). As shown in **Figure 4D**, compared with control WT mice, the level of serum UA was significantly elevated on 3 dpi in *Nlrc3^−/−^* mice, serum CK-MB was significantly elevated on 9 dpi in *Nlrc3^−/−^* mice, but serum levels of ALT, UREA, and CREA had shown no elevation (**Supplementary Table 1**).

Renal involvement in HFRS infected with PUUV includes transient proteinuria, microscopic but rarely visible hematuria, and oliguric AKI, followed by polyuria and recovery. The typical feature of proteinuria in this disease was its rapid decrease after the acute phase of the disease (6). In a large cohort of patients with acute tubulointerstitial nephritis caused by various etiologies, proteinuria was found in only one-fourth of the patients, while only 2% of all patients, nephrotic range proteinuria was found (28). We collected the urine on 0, 3, 6 dpi and found the urine protein levels increased on 3 dpi, the acute phase of infection. The urine protein concentration was significantly increased and the average value was close to 0.3 g/L at 3 dpi in the infected *Nlrc3^−/−^* mice and WT mice (**Figure 4E**).

**Figure 5:**
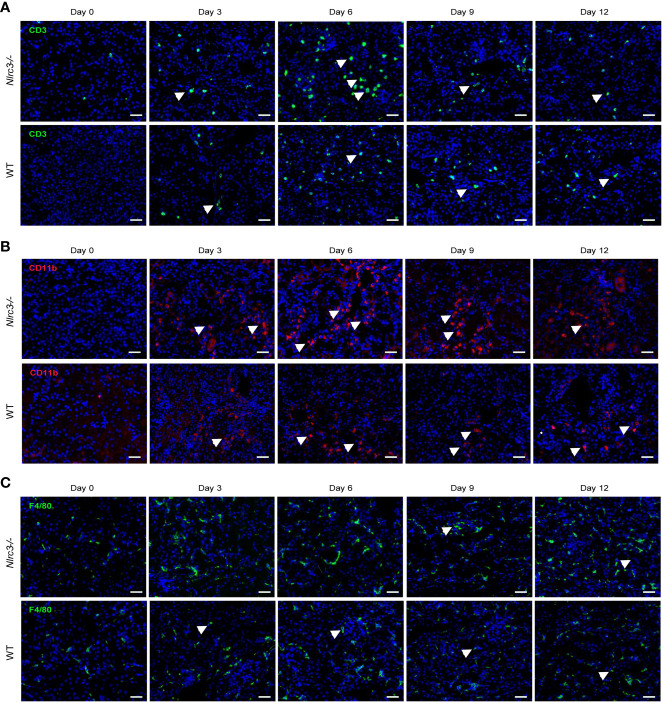
IHC staining for inflammatory cell infiltration in the kidney of HTNV infected mice. **(A)** A representative image of IHC assay and CD3 was used to detect the infiltration of inflammatory T cells. The upper panel shows the kidney of *Nlrc3^−/−^* mice; the lower panel shows the kidney of WT mice. Green signals represent CD3, and blue indicates cell nucleus. **(B)** Representative image of IHC staining for CD11b of the kidney of HTNV infected mice. CD11b was used to detect myeloid cells, and Upper panel shows the kidney of *Nlrc3^−/−^* mice; the lower panel shows the kidney of WT mice. Red signals represent CD11b; Blue signals represent DAPI. **(C)** Representative image of IHC staining for F4/80 of the kidney of HTNV infected mice. F4/80 was used to detect macrophages. The upper panel shows the kidney of *Nlrc3^−/−^* mice; the lower panel shows the kidney of WT mice. Green signals represent F4/80; Blue signals represent DAPI. IHC images are representative of at least three samples. Scale bars, 50 μm.

To examine the inflammatory cells infiltrated into renal interstitial after HTNV infection, we performed immunohistochemistry (IHC) staining with CD3, F4/80, and CD11b. The results showed that a group of immune cells positive for CD3 have infiltrated into the renal interstitial after HTNV infection (**Figure 5A**), and myeloid cells positive for CD11b appeared in the cortical tubulointerstitial (**Figure 5B**). The proportion of infiltrating CD3^+^ T cells and CD11b^+^ myeloid cells is significantly higher in infected *Nlrc3^-/-^* mice than WT mice, but macrophages positive for F4/80 distributed all over the renal cortex and medulla and showed no significant difference between these two mouse types (**Figure 5C**).

### HTNV Infection Induced a Robust Cytokine Response in *Nlrc3^−/−^* Mice

The overproduction of inflammatory cytokines is commonly reported in subjects with HFRS and has given rise to the hypothesis that cytokine dysregulation may play a pivotal role in the pathogenesis of the disease. Vaheri et al. (8) found that multiple cytokines were produced by various cells, such as macrophages, monocytes, and lymphocytes, in response to pro-inflammatory signals such as viral infection. Wang et al. reported that the serum concentrations of TNF-α, IL-6, IFN-γ, IL-8, IP-10, and RANTES (but not IL-4) were much elevated in HFRS patients (29), compared with controls; and that the highest concentrations were usually found during the febrile, hypotensive, and oliguric phases, particularly in severe and critical-type HFRS cases. High plasma IL-6 levels were associated with severe renal failure and thrombocytopenia in PUUV-induced HFRS and could be used as a marker of disease severity.

**Figure 6:**
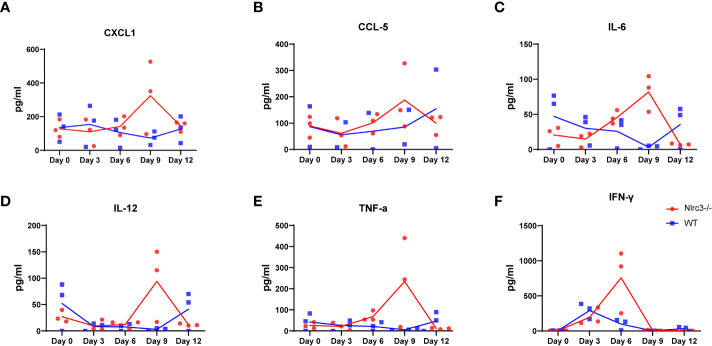
Inflammatory Response in HTNV-infected Mice. The serum of each mice was collected on 3, 6, 9, and 12 dpi for cytokine measurement. The levels of CXCL1 **(A)**, CCL5 **(B)**, IL-6 **(C)**, IL-12 **(D)**, TNF-α **(E)** IFN-γ **(F)** in serum of HTNV-infected mice (pg/mL). Red dots and lines represent *Nlrc3^−/−^* mice, and blue indicate WT mice. Bars are means ± SD. *Nlrc3^−/−^* mice, n=3; WT mice, n=3.

Our results showed that HTNV infection led to elevated cytokine production, including CXCL-1, CCL-5, IL-6, IL-12, TNF-α, and IFN-γ in *Nlrc3^−/−^* mice, and the levels of these cytokines peaked at 6 or 9 dpi; in comparison, WT mice had weaker cytokine responses throughout the infection process (**Figure 6**).

In conclusion, we found in this study that after intraperitoneal inoculation of HTNV, *Nlrc3^−/−^* mice develop weight loss, renal hemorrhage, and tubule dilation. These features resemble clinical symptoms of HFRS more closely than any other previously published animal models. Moreover, the *Nlrc3^−/−^* mice also showed a higher viral load in the serum, spleen, and kidney than WT mice, and *Nlrc3^−/−^* mice developed hematological disorder and significant pathological changes in multiple organs, and thus establishing HTNV infection of *Nlrc3^−/−^* mice as a new model for studying HFRS.

## Discussion

The HFRS mouse model described here demonstrates that HTNV infected *Nlrc3^−/−^* mice represent a suitable small animal model that may be used to evaluate HFRS vaccines and therapeutics. The *Nlrc3^−/−^* mouse model resembles systematic injury observed in humans, characterized by thrombocytopenia, lymphocytopenia, elevated serum levels of AST (liver dysfunction), LDH, creatine kinase MB fraction, and UA (renal dysfunction). Furthermore, the *Nlrc3^−/−^* mice developed weight loss and apparent renal hemorrhage. Viral replication and histopathological alterations in target organs were detected in the lung, spleen, liver, and kidney. In addition, a modestly higher level of viral RNA loads was found in the blood, spleen, and kidney of *Nlrc3^−/−^*mice. In comparison, WT mice developed mild histological changes despite detecting viral RNA in the blood, spleen, and kidney.

Our findings are generally in agreement with what would be expected for HTNV infection-induced HFRS and signify an improvement over previously published animal models. It has been reported that hemorrhagic fever (HF) virus infection in animal models can lead to a spectrum of diseases from asymptomatic to mild and severe and often transient viremia. A transient weight loss usually manifests mild illness, but severe disease showed more significant weight loss (> 20%) (30). For example, hamsters infected with HTNV *via* nasal route led to a persistent asymptomatic infection, characterized by sporadic viremia but high levels of viral genome copies in the lungs, brain, and kidneys. Ferrets infected with high dose HTNV *via* muscle injection showed gradual bodyweight decrease (5-12% over time) but intact renal functions, absence of viremia, and lack of virus transmission to organs. In marmosets, intramuscular injection of HTNV led to a high degree of seroconversion and production of neutralizing antibodies; consistently, these animals had no kidney damage, viremia, or virus transmission to organs (13).

As to the observation that hematological changes only observed at a single time point and cytokine alterations are not significant, we think they can be explained by the nature of HTNV infection, which is similar to many other viral infections that afflict multiple organs, and therefore often difficult to find sustained changes of a single parameter. Overall, our findings are generally in line with the existing knowledge in the field. Furthermore, the discovery of *Nlrc3^−/−^* mice having more susceptibility to HTNV infection and showing many changes similar to the pathological changes of HFRS is a new contribution to the field.

It is generally accepted that human vascular endothelial cells are the primary targets for hantavirus, which leads to variable degrees of generalized capillary dilatation and edema (31). Viral antigens could be found within the capillary endothelium of various tissues (Hantaviruses direct endothelial cell permeability by sensitizing cells to the vascular permeability factor VEGF, while angiopoietin 1 and sphingosine 1-phosphate inhibit hantavirus-directed permeability). Histological analysis showed that the kidney of *Nlrc3^−/−^* mice were observed renal tubules dilatation at 9 dpi, *Nlrc3^−/−^* mice with more severe pathological changes than WT mice and had persisted on 12 dpi. In *Nlrc3^−/−^* mice, renal tubular epithelial cell edema and remarkable renal interstitial hemorrhage were observed in the kidney. Further, prominent extra-medullary hematopoiesis in the red pulp of the spleen was observed on 6, 9, and 12 dpi, and germinal center dilations and an increased number of multinucleated giant cells on 12 dpi. The livers also showed severe hepatocytic degeneration and scattered necrosis on days 3, 6, 9, and 12 dpi. Lung inflammation and alveolar wall thickening were observed on 6 dpi. This finding suggests that HTNV infection had a significant and long-term impact on the liver and kidney in HTNV-infected *Nlrc3^−/−^* mice. In addition, *Nlrc3^-/-^* mice showed prominent renal tubule dilation, interstitial hemorrhage, and renal dysfunction after HTNV infection. The level of serum UA was significantly elevated on 3 dpi, a clear indication of renal dysfunction. The urine protein concentration was significantly increased and the average value was close to 0.3 g/L at 3 dpi in the infected *Nlrc3^-/-^* mice. The same trend of UA increase was not observed in WT mice. These results suggest our model recapitulates more pathological features of human HTNV infection.

The N protein (NP) is produced abundantly in hantavirus infected cells and can be detected early as 4 h post-infection (32). These features account for the main reason why the endpoint of ELISA is to detect hantavirus N protein and immunofluorescence assays for assessing the number of viral particles and the progression of viral infection. Different methods may affect results obtained, and conclusions reached. Viral protein and viremia are two different parameters of viral infection, and they may not always change synchronously. The results of quantitative real-time PCR showed that a transient elevation of NP levels occurred on 6 dpi in the spleen, where the virus might have undergone a transient replication, followed by its clearance after that. As expected, viral protein accumulation was observed after the peak viremia, which appeared on 3 dpi. Indeed, viral replication in the spleen and kidney maintained at a moderate level on 9 and 12 dpi, accompanied by N protein synthesis (spleen at 9 dpi: WT mice:192 ± 128 copies per mg; *Nlrc3^−/−^* mice: 431 ± 229 copies per mg; spleen at 12 dpi: WT mice:160 ± 85 copies per mg; *Nlrc3^−/−^* mice: 226 ± 118 copies per mg). However, the amount of HTNV NP at protein level reached the maximum on day 6 p.i. and stay elevated throughout 9 and 12 dpi. The difference in the kinetics of viral RNA copies and protein amount might reflect their differential decay in different organs.

The role of cytokines is usually more complex in disease models. In PUUV-induced HFRS, severe renal failure and thrombocytopenia are associated with high plasma IL-6 levels (33). Our analysis showed that HTNV infection led to elevated cytokine production, including CXCL-1, CCL-5, IL-6, IL-12, TNF-α and IFN-γ in *Nlrc3^−/−^* mice, but not as much in WT mice. It is worth noting that at 9 dpi, *Nlrc3^−/−^* mice showed remarkable pathological changes in their spleen and kidney, accompanied by a robust cytokine response at this time. These findings indicate that a high level of cytokines might be related to the pathogenesis of the disease.

In summary, the results we report here demonstrate that after inoculated with HTNV through the intraperitoneal route, *Nlrc3^−/−^* mice develop weight loss, thrombocytopenia, renal dysfunction, and hemorrhage. These resemble more closely the clinical symptoms of HFRS than any other previously published animal models. This model can be used to evaluate potential vaccines and therapeutics and in-depth study of HFRS pathogenesis *in vivo*.

## Data Availability Statement

The original contributions presented in the study are included in the article/**Supplementary Material**. Further inquiries can be directed to the corresponding authors.

## Ethics Statement

The animal study was reviewed and approved by Committee of Laboratory Experimentation of Fourth Military Medical University Animal Center (No.20210403).

## Author Contributions

RL and RM designed the study. XZ and ZL performed viral infections. WS and SH collected mouse sera. YL and QY recorded mouse body weight and temperature. RM was responsible for western blot and qRT-PCR assay. RM was responsible for data summary and data analysis. JS and FW performed the statistical analysis. RL and RM drafted the manuscript. XJ and XW critically revised the manuscript. All authors contributed to the article and approved the submitted version.

## Funding

This work was supported by the National Science Foundation (Nos. 81772167, 81971563) and the Key Research and Development Project of Shaanxi Province (No. 2019ZDLSF02-03).

## Conflict of Interest

The authors declare that the research was conducted in the absence of any commercial or financial relationships that could be construed as a potential conflict of interest.
