# Supplementary material for: Nlrc3 Knockout Mice Showed Renal Pathological Changes After HTNV Infection
Source: Front Immunol. 2021 Jul 16;12:692509. doi: 10.3389/fimmu.2021.692509 (PMC8322986; doi:10.3389/fimmu.2021.692509)
Supplement: Supplementary file 1 [file DataSheet_1.docx]

Supplementary Material

## Supplementary Figures


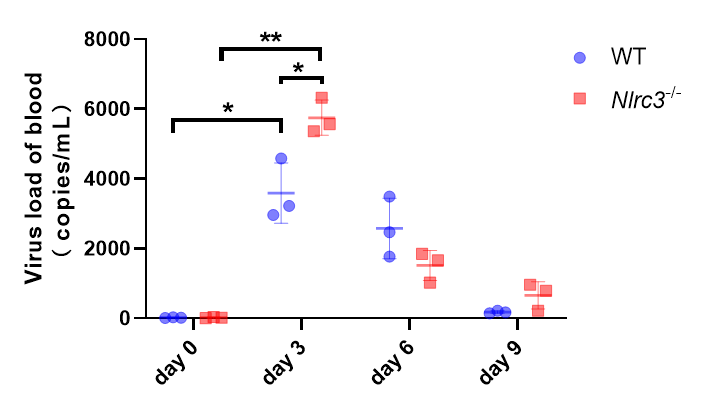


**Supplementary Figure 1.** EDTA**-**anticoagulation of whole blood was collected at 3, 6, and 9, and 12 dpi. Viral loads in the whole blood of HTNV-infected mice were measured on (*Nlrc3^−/−^* mice, n=3, WT mice, n=3) by real-time PCR. Bars represented means ± SD. Asterisks indicate a significant difference in the quantity of viral RNA loads (*p*<0.05).


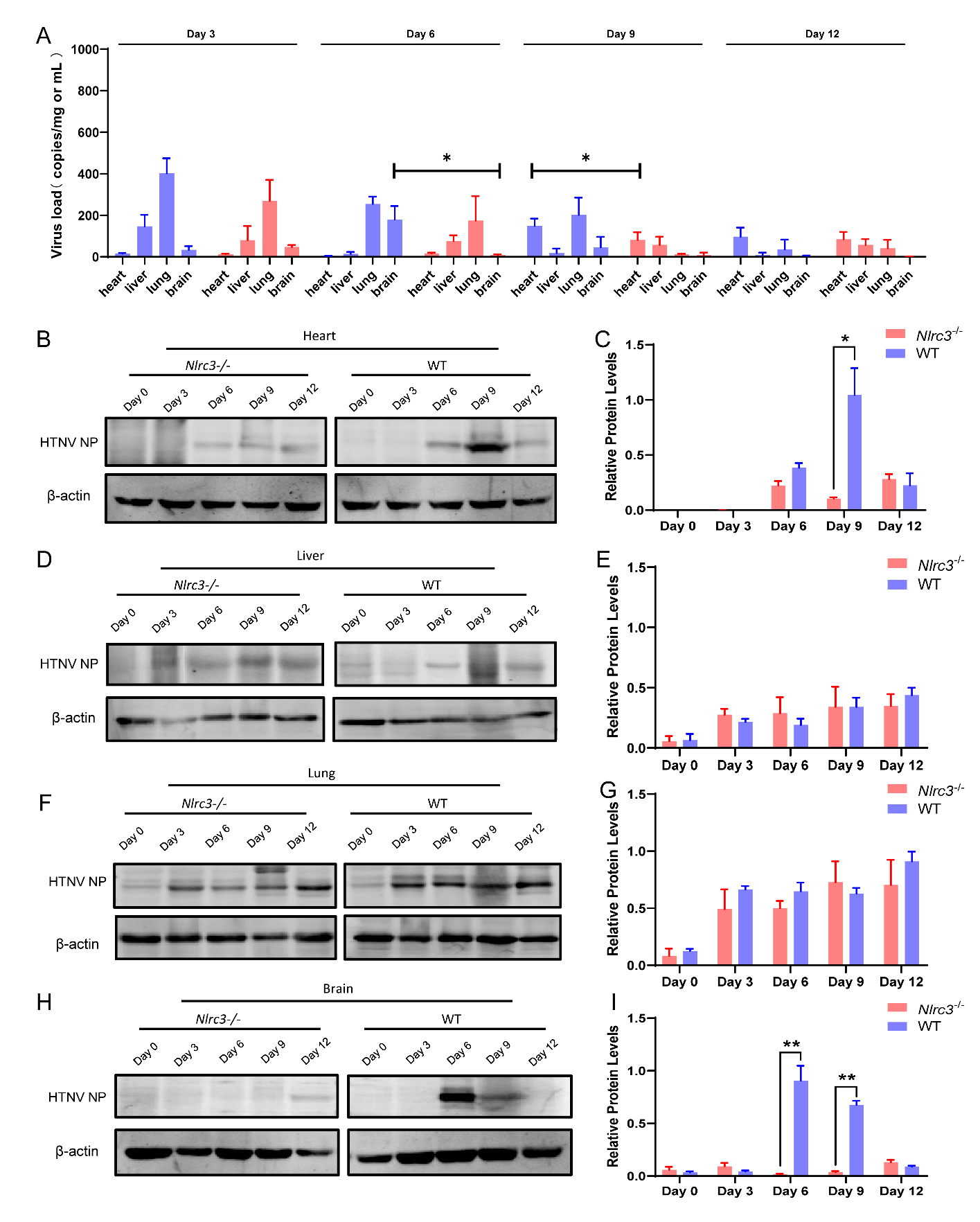


**Supplementary Figure 2.** Levels of viral replication in the heart, liver, lung, and brain of HTNV-infected mice. (A) Viral loads in each organ were measured by qRT-PCR. (B) Western blot results of the heart of *Nlrc3^−/−^* and WT mice. Lanes represent corresponding time points. (C) Densitometric analysis of band intensity for B. (D) Western blot results of the liver. (E) Densitometric analysis of band intensity for D. (F) Western blot results of the brain. (G) Densitometric analysis of band intensity for F. (H) Western blot results of the lung. (I) Densitometric analysis of band intensity for H (*Nlrc3^−/−^*, n=25; WT mice, n=20). Bars are means ± SD.


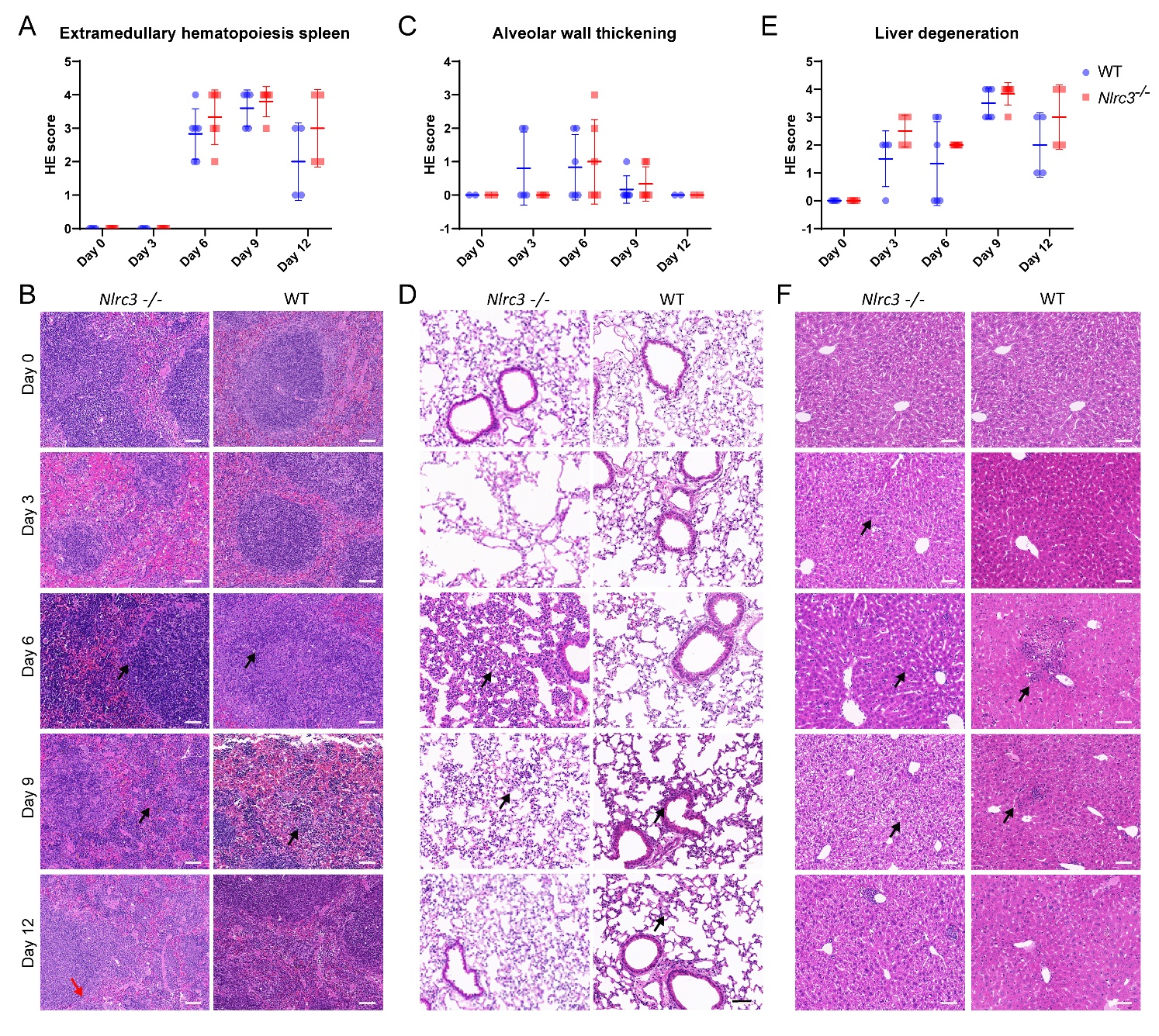


**Supplementary Figure 3.** Pathological changes in the spleen, lung, and liver of HTNV-infected mice. The spleen, lung, and liver were collected at 3, 6, 9, and 12 dpi, and then fixed in 4% paraformaldehyde, followed by H&E staining. (A) Statistical figure based on histological scores of extra-medullary hematopoiesis of the spleen for *Nlrc3^−/−^* mice (n=6) and WT mice (n=6). (B) The representative image of spleen from *Nlrc3^−/−^* and WT mice infected with HTNV showing prominent extra-medullary hematopoiesis. (C) Statistical figure based on histological scores of alveolar walls thickening of the lung for *Nlrc3^−/−^*mice (n=6) and WT mice (n=6). (D) The representative image of the lung from *Nlrc3^−/−^* and WT mice infected with HTNV showing severe alveolar wall thickening. (E) Statistical figure based on histological scores of degenerations of hepatocytes of the liver for *Nlrc3^−/−^* mice (n=6) and WT mice (n=6). (F) The representative image of liver from *Nlrc3^−/−^* and WT mice infected with HTNV showing ballooning degeneration of hepatocytes and scattered necrosis. H&E images are representative of at least three samples. Scale bars, 50 μm.

**Supplementary Table 1. Serum biochemistry profile of mice after infection of HTNV**

|  | Days post infection | | | | | | | | | | |
| --- | --- | --- | --- | --- | --- | --- | --- | --- | --- | --- | --- |
| Tests (unit) | Day 0 | | Day 3 | | Day 6 | | Day 9 | | Day 12 | | |
| **Parameter** | **WT** | **KO** | **WT** | **KO** | **WT** | **KO** | **WT** | **KO** | **WT** | **KO** |  |
| AST (U/L) | 144.91±19.93 | 136.5±31.82 | 152.92±72.24 | 162.27±52.69 | 198.06±40.78 | 186.86±35.39 | 162.29±39.8 | 191.65±92.96 | 159.4±81.7 | 131.78±25.98 |  |
| ALT (U/L) | 61.96±2.27 | 62.08±2.44 | 34.10±5.6 | 53.12±12.86 | 70.3±8.08 | 68.06±10.72 | 56.7±9.63 | 62.48±14.63 | 53.43±4.4 | 47.79±10.37 |  |
| LDH (U/L) | 483.16±92.77 | 467.84±114.44 | 301.9±12.69 | 1024.28±339.48* | 484.18±16.13 | 489.08±104.39 | 437.34±93.72 | 610.04±326.27 | 385.41±54.64 | 414.78±65.1 |  |
| UREA (mM) | 27.98±7.11 | 26.25±4.66 | 19.21±2.53 | 25.91±8.7 | 21.68±6.65 | 26.83±3.68 | 30.26±5.41 | 26.32±5.63 | 22.04±2.6 | 28.92±3.68 |  |
| CREA (μM) | 16.59±7.87 | 19.47±3.79 | 19.56±5.29 | 15.92±4.89 | 11.45±4.71 | 10.21±3.65 | 11.94±5.04 | 9.09±6.48 | 12.44±6.15 | 11.01±4.15 |  |
| CK-MB (U/L) | 137.41±48.5 | 134.51±44.4 | 125.81±17.62 | 142.56±31.09 | 142.02±43.06 | 141.29±69.53 | 133.72±34.39 | 198.75±18.04* | 130.73±8.13 | 86.95±17.57 |  |

Data are mean±SD. AST is short for aspartate transaminase. ALT is short for alanine transaminase. LDH is short for lactate dehydrogenase. UREA means urea. CREA means creatinine. CK-MB means Creatine Kinase-MB.

* Asterisks indicate a significant difference between the two groups (*p*<0.05).
